# 3D clusters of somatic mutations in cancer reveal numerous rare mutations as functional targets

**DOI:** 10.1186/s13073-016-0393-x

**Published:** 2017-01-23

**Authors:** Jianjiong Gao, Matthew T. Chang, Hannah C. Johnsen, Sizhi Paul Gao, Brooke E. Sylvester, Selcuk Onur Sumer, Hongxin Zhang, David B. Solit, Barry S. Taylor, Nikolaus Schultz, Chris Sander

**Affiliations:** 1 Marie-Josée and Henry R. Kravis Center for Molecular Oncology, Memorial Sloan Kettering Cancer Center, New York, NY USA; 2 Human Oncology and Pathogenesis Program, Memorial Sloan Kettering Cancer Center, New York, NY USA; 3 Department of Epidemiology and Biostatistics, Memorial Sloan Kettering Cancer Center, New York, NY USA; 4 Departments of Bioengineering and Therapeutic Sciences, University of California, San Francisco, CA USA; 5 Louis V. Gerstner Jr. Graduate School of Biomedical Sciences, Memorial Sloan Kettering Cancer Center, New York, NY USA; 6 Department of Medicine, Memorial Sloan Kettering Cancer Center, New York, NY USA; 7 Weill Cornell Medical College, Cornell University, New York, NY USA; 8 Department of Cell Biology, Harvard Medical School, Boston, MA USA; 9 cBio Center, Dana-Farber Cancer Institute, Boston, MA USA; 10 Computational Biology Program, Memorial Sloan Kettering Cancer Center, New York, NY USA

**Keywords:** Cancer genomics, Driver mutations, Protein structures, Precision medicine

## Abstract

Many mutations in cancer are of unknown functional significance. Standard methods use statistically significant recurrence of mutations in tumor samples as an indicator of functional impact. We extend such analyses into the long tail of rare mutations by considering recurrence of mutations in clusters of spatially close residues in protein structures. Analyzing 10,000 tumor exomes, we identify more than 3000 rarely mutated residues in proteins as potentially functional and experimentally validate several in RAC1 and MAP2K1. These potential driver mutations (web resources: 3dhotspots.org and cBioPortal.org) can extend the scope of genomically informed clinical trials and of personalized choice of therapy.

**Electronic supplementary material:**

The online version of this article (doi:10.1186/s13073-016-0393-x) contains supplementary material, which is available to authorized users.

## Background

Recent large-scale sequencing efforts such as The Cancer Genome Atlas (TCGA) have revealed a complex landscape of somatic mutations in various cancer types [1]. While the data generated have provided a more complete picture of the genomic aberrations in cancer cells, the interpretation of individual mutations can be difficult. One of the key challenges is distinguishing the few mutations that functionally contribute to oncogenesis (“drivers”) from the many biologically neutral mutations (“passengers”) [2].

Several methods are currently being used to identify driver genes based on the frequency of mutations observed in a gene across a set of tumors, e.g., MutSig [3] and MuSiC [4]. These methods have two limitations: (1) their unit of analysis is a gene and they do not distinguish individual driver mutations from passengers in a given gene, and (2) they are not able to detect functional mutations in infrequently mutated genes, often referred to as the “long tail” of the frequency distribution of somatic mutations in cancer [5].

To move beyond a gene-level definition of drivers and to identify position- and allele-specific driver mutations, we previously developed a statistical method that identified hundreds of single-residue mutational hotspots across various cancer types [6]. However, the vast majority of somatic mutations identified in tumors occur infrequently and most are likely non-functional passenger events. But a small subset of these rare mutations represent functional driver events, and these would be overlooked by methods that rely exclusively on mutation frequency at individual amino acid positions. It is therefore important to develop more refined methods that at the genome scale identify infrequent mutations that are likely functional. Though individually rare, these long-tail mutations are present in a significant fraction of tumors and are likely key molecular events and thus potential drug targets [5]. Several methods exist that identify driver genes or mutations in the long tail by incorporating protein-level annotation, such as local positional clustering [7], phosphorylation sites [8], and paralogous protein domains [9].

Recently, three-dimensional (3D) protein structures have also been used to identify driver genes and mutations in cancer and other diseases. For example, Dixit et al. [10] studied cancer mutations in 3D structures of protein kinases. Wang et al. [11] generated a structurally solved interactome to study genetic diseases. Porta-Pardo et al. [12] and Engin et al. [13] used 3D structures to detect protein-protein interaction interfaces that are enriched with cancer mutations. Clustering of mutations in protein structures (CLUMPS) [14] used 3D clustering of mutations to detect cancer genes and also studied enrichment of mutations in protein-protein interaction interfaces. StructMAn [15] annotated the amino acid variations of single-nucleotide polymorphisms (SNPs) in the context of 3D structures. SpacePAC [16], Mutation3D [17], HotMAPS [18], and Hotspot3D [19] used 3D structures to identify mutational clusters in cancer. These efforts have generated interesting sets of candidate functional mutations and illustrate that many rare driver mutations are functionally, and potentially clinically, relevant.

**Fig. 1:**
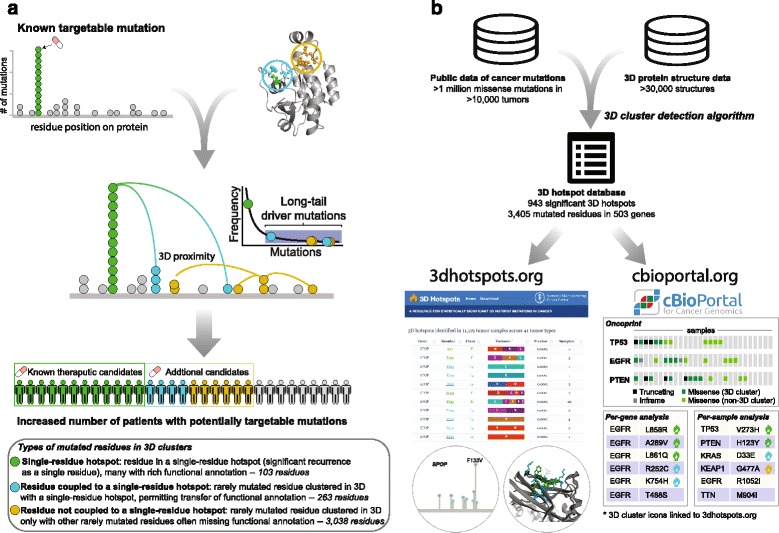
Mutational 3D cluster analysis method and related resources. **a** Process of going beyond single-residue hotspots by considering occurrence in 3D clusters. The colors of different types of mutated residues in 3D clusters are defined in the *bottom panel* and used throughout the manuscript. **b** Mutations in 3D clusters can be explored via the web resource http://3dhotspots.org. The results are also made available via a web API service for use by other bioinformatics tools, and mutations viewed in the cBioPortal for Cancer Genomics are annotated if they are part of an identified 3D cluster. The identified 3D clusters are likely to change as the cancer genomics and 3D structure databases grow

Here, we describe a novel method that identifies mutational 3D clusters, i.e., missense (amino-acid-changing) mutations that cluster together in 3D proximity in protein structures above a random background, with a focus on identifying rare mutations. In this largest 3D cluster analysis of whole exome or genome sequencing data in cancer to date, we analyzed more than one million somatic missense mutations in 11,119 human tumors across 32,445 protein structures from 7390 genes. The analysis identified potential driver mutations, the majority of which are rare mutations (occurring in <0.1% of patients in the dataset), in 3405 residues clustering in the protein structures of 503 genes (Fig. 1). Many of these 3D clusters were identified in well-characterized cancer genes, such as *KRAS*, *BRAF*, and *TP53*, and include known oncogenic recurrent alleles (e.g., KRAS G12D) as well as rare long-tail alleles (e.g., KRAS D33E, which has recently been experimentally validated [20]). We were able to identify new potential driver genes as well as novel candidate driver mutations in clinically actionable cancer genes that were not detected by our mutational single-residue hotspot detection method [6] and other 3D cluster detection methods [17–19]. We experimentally tested the activating potential of rare mutations identified in 3D clusters in the MAP2K1 and RAC1 proteins, enlarging the number of biologically and potentially clinically significant alleles in these two critical effectors of activated signaling pathways in cancer. To facilitate further biological and clinical validation, we have made the catalog of 3D cluster mutations available through an interactive web resource (http://3dhotspots.org) and in the widely used cBioPortal for Cancer Genomics (http://cbioportal.org) [21, 22] (Fig. 1b).

## Methods

### Mutational data collection and processing

Mutational data were obtained from publicly available sources including The Cancer Genome Atlas (TCGA), the International Cancer Genome Consortium (ICGC), and published studies from the literature [21, 22]. Mutations were processed as described previously [6]. Briefly, genomic coordinates of variants were standardized to the human reference assembly GRCh37. Genomic coordinates from previous assemblies were converted to GRCh37 via LiftOver (https://genome.ucsc.edu/cgi-bin/hgLiftOver). Mutations were annotated based on Ensembl release 75, and the mutational effect was annotated on canonical isoforms per gene defined by UniProt canonical sequences (http://www.uniprot.org/help/canonical_and_isoforms) using Variant Effect Predictor (VEP) version 77 (http://ensembl.org/info/docs/tools/vep/) and vcf2maf version 1.5 (https://github.com/mskcc/vcf2maf). To remove potential germline variants misreported as somatic mutations, we excluded mutations found in both the 1000 Genomes Project and the National Heart, Lung, and Blood Institute (NHLBI) Exome Sequencing Project, as well as those identified in the 1000 Genomes Project in two or more samples. Furthermore, we removed mutations in genes whose RNA expression was less than 0.1 transcript per million (TPM) in 90% or more of the tumors of that type based on TCGA RNA expression data. For samples whose cancer types lack RNA expression data, genes were removed if more than 95% of all tumors in our dataset had RNA expression of TPM less than 0.1. Complete details on data processing were documented in Chang et al. 2016 [6].

### Protein 3D structure data collection and processing

Protein structures were downloaded from the Research Collaboratory for Structural Bioinformatics (RCSB) Protein Data Bank (PDB, http://www.rcsb.org/) [23]. Alignments of protein sequences from UniProt [24] to PDB were retrieved from MutationAssessor [25] and the Structure Integration with Function, Taxonomy and Sequences (SIFTS) resource [26]. Only alignments with a sequence identity of 90% or above were included. For each structure chain, a contact map of residues was calculated. Two residues are considered in contact if any pair of their atoms is within 5 angstroms (Å), as calculated by BioJava Structure Module [27]. A 3D cluster is defined by a central residue and its contacting neighbor residues (Additional file 1: Figure S1a). All residues are used in turn as centers of clusters. The test of statistical significance (described in the following subsection) is applied separately to each cluster in turn. Clusters are not merged, so each residue can be in more than one cluster, even after filtering for statistical significance of the clusters.

### Identifying significantly mutated 3D clusters

A 3D cluster was identified as significantly mutated if its member residues were more frequently mutated in the set of samples than expected by chance. Mutations were mapped to the aligned PDB sequences and structures (Additional file 1: Figure S1a), and the total number of mutations across all samples was calculated within each 3D cluster. To determine whether the residues in a 3D cluster in a particular structure were more frequently mutated than expected by chance, a permutation-based test was performed by generating 10^5^ decoy mutational patterns on the aligned region of the protein structure. A decoy pattern was generated by randomly shuffling the residue indices (positions in the sequence), with their associated mutation count, on the structure (Additional file 1: Figure S1b, c). For each decoy mutational pattern, the number of mutations in each cluster was calculated as above. For a given 3D cluster in question, the *p* value was calculated as the fraction of decoys for which the number of mutations (based on the decoy data) in any cluster was equal to or larger than the number of mutations (based on the real data) in the 3D cluster in question. When shuffling the mutations, the mutation count in each residue was maintained, except that we set the maximum number of mutations in one residue in the decoy to the largest number of mutations in the assessed 3D cluster with the intent of ensuring detection of less frequently mutated 3D clusters within a gene with one or a few dominant single-residue hotspots (such as BRAF V600) (Additional file 1: Figure S1b, c). In the rest of the manuscript, we use the term ”3D cluster” as a short alias for ”significantly mutated 3D cluster.”

### Experimental assays to test identified MAP2K1/MEK1 mutations

#### Cell line and culture

Human embryonic kidney HEK-293H cells (Invitrogen) were maintained in Dulbecco's Modified Eagle's (DME)-HG medium with 10% fetal bovine serum (FBS), supplemented with 2 mM glutamine, and 50 units/ml each of penicillin and streptomycin.

#### Transfections

MAP2K1 mutant constructs were generated from the MEK1-GFP plasmid (#14746, Addgene, Cambridge, MA, USA) using the QuikChange II XL Site-Directed Mutagenesis Kit (Stratagene) as recommended. All mutant plasmids were verified by Sanger sequencing. HEK-293H cells were seeded for 70–90% confluency at the time of transfection, then transiently transfected with the wild-type or mutant MEK1-GFP plasmid using Lipofectamine® 2000 Transfection Reagent (Invitrogen). Plasmid transfection levels were standardized according to green fluorescent protein (GFP) expression. Cells were collected 24 hours post-transfection.

#### Western blot analysis

Cells were lysed in 1% NP-40 buffer with protease and phosphatase inhibitors, then processed for immunoblotting as previously described [28]. Rabbit polyclonal antibodies recognizing MEK1/2, phosphorylated ERK1/2 (Thr202/Tyr204), and ERK1/2 were obtained from Cell Signaling, Danvers, MA, USA. Rabbit monoclonal antibodies recognizing GFP and GAPDH were obtained from Cell Signaling. After incubation with horseradish peroxidase-conjugated secondary antibody, proteins were detected by chemiluminescence (SuperSignal West Dura Chemiluminescent Substrate, Thermo Scientific) and visualized using the Fuji LAS-4000 imager (GE Life Sciences, Pittsburgh, PA, USA).

#### Drug experiments

HEK-293H cells were transfected with MEK1 wild-type or mutant GFP-tagged plasmid. At 24 hours, cells were treated with 100 nM trametinib (Selleck Chemicals, Houston, TX, USA) and collected after 2 hours. Control cells were treated with dimethyl sulfoxide (DMSO). Cells were lysed for protein and immunoblotted as referenced above.

### Experimental assay to test identified RAC1 mutations

#### Cell line and culture

Early-passage HEK-293 T cells, acquired from American Type Culture Collection (ATCC), Manassas, VA, USA and authenticated as mycoplasma free, were cultured at 37 °C in 5% CO_2_ in DMEM supplemented with 10% FBS.

#### Transfections

RAC1 mutation validation was performed similarly to what was previously described [6]. DNA coding sequences for mutant RAC1 constructs were generated via site-directed mutagenesis (Genewiz, South Plainfield, NJ, USA). All mutant plasmids were verified by Sanger sequencing. RAC1 constructs contained an N-terminal 3xFLAG epitope tag and were subcloned into a pcDNA3 mammalian expression vector (Life Technologies, Grand Island, NY, USA). The expression constructs were transfected into these cells using Lipofectamine 2000 (Life Technologies).

#### Western blot analysis

Cells were harvested 72 hours after transfection. GTP-bound RAC1 (active RAC1) was isolated via immunoprecipitation using recombinant p21-binding domain (PBD) of PAK1 (PAK1-PBD; Active RAC1 Detection Kit, Cat. #8815, Cell Signaling Technology), according to the manufacturer's instructions. Total RAC1 was detected using kit-provided RAC1 primary antibody.

## Results

### A catalog of mutational clusters in protein structures

**Fig. 2:**
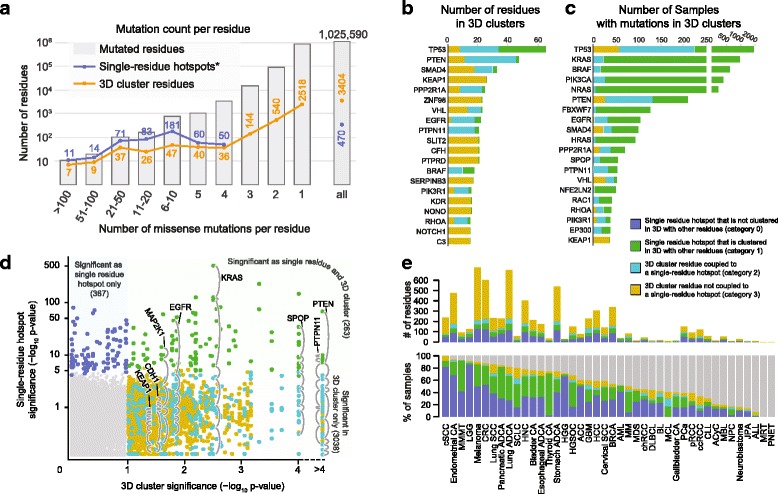
3D cluster analysis reveals numerous potentially functional rare mutations. **a** 3D cluster analysis identified a large number of statistically significant, yet rarely mutated residues (mutated one to three times in our dataset). The residues were binned by the number of mutations in each residue. The mutation counts for the single-residue hotspots also contain a small fraction of silent, nonsense, and splice-site mutations identified by Chang et al. 2016 [6]. **b** Genes with the highest number of residues in 3D clusters. **c** Genes with the highest frequency of tumor samples with mutations clustered in 3D structures across all cancer types. **d** Per-residue comparison of significance as in single-residue hotspot (*vertical axis*) and 3D cluster (*horizontal axis*). Many residues were hotspots as well as parts of 3D clusters (*upper right quadrant*), but some were detected only as part of 3D clusters (*bottom right quadrant*). **e** Number of residues (*upper panel*) and percentage of samples (*bottom panel*) with hotspots and 3D clusters per cancer type (see full cancer type names in the Abbreviations section). The category of a sample was assigned based on the lowest category if it had mutations that belonged to different categories

We have curated a comprehensive dataset of somatic mutations, consisting of sequenced exomes and genomes of 11,119 human tumors spanning 41 cancer types. The dataset contained 1,182,802 somatic missense mutations occurring in 1,025,590 residues in 18,100 genes, out of which the protein sequences of 7390 genes were aligned to 32,445 protein 3D structures. Most (908,009) of these residues were mutated only once in the 11,119 samples (Fig. 2a); i.e., most somatic mutations found in cancer are extremely rare. Most of these rare mutations are likely passenger mutations, but some may be unrecognized drivers [20]. Indeed, we found that a small fraction of rarely mutated residues (e.g., mutated in three or fewer samples) are members of recurrently mutated clusters in 3D structures (Fig. 2a) and thus probably are functional drivers.

In total, we identified 943 unique mutational clusters (clusters with the same set of residues in amino acid sequence were counted as one unique cluster) that were statistically significant in 2382 protein structures (Additional file 2: Table S1). These 3D clusters encompassed 3404 residues in 503 genes (Additional file 3: Table S2). *TP53* contained the largest number of residues in 3D clusters (66 residues), followed by *PTEN* (48), *SMAD4* (33), and *KEAP1* (26) (Fig. 2b, Additional file 4: Table S3). *TP53* mutations in 3D clusters were also the most prevalent across all cancer types (in 1914 samples, 17%), followed by *KRAS* (8%), *BRAF* (6%), and *PIK3CA* (4%), underscoring the roles of these well-characterized cancer genes in oncogenesis (Fig. 2c, Additional file 5: Table S4).

We classified the mutated residues in a 3D cluster into three categories (Figs. 1 and 2d, Additional file 3: Table S2) depending on whether the cluster contains single-residue hotspots identified by [6]: (1) 103 residues in single-residue hotspots, (2) 263 rarely mutated residues that were clustered in 3D with a single-residue hotspot, and (3) 3038 rarely mutated residues that were clustered in 3D only with other rarely mutated residues. If a rarely mutated residue belonged to category 2 in one cluster and category 3 in another, the residue was classified as category 2. There were 367 hotspots identified by [6] that were not detected in 3D clusters (Fig. 2d), either because they were not part of a significant cluster with other mutated residues or because there was no 3D structure available for the protein or protein region.

Notably, in 5038 samples (45%), prior frequency-based hotspot analysis failed to identify single-residue hotspot driver mutations. By incorporating protein structure data, rare mutations present in 3D clusters were identified in 865 of these samples (17% of the samples without single-residue hotspot driver mutations, or 8% of all samples) (Fig. 2e). As an example, 141 (15%) of 961 lung tumors (lung adenocarcinoma, lung squamous cell carcinoma, and small-cell lung cancer) with no single-residue hotspot mutations carried a rare mutation in a 3D cluster. Assuming the diseases of these patients were genetically driven, these 3D cluster mutations were possibly driver events (Fig. 2e).

### 3D cluster analysis identified rare missense driver mutations in tumor suppressor genes

While tumor suppressor genes are often inactivated by truncating (e.g., nonsense and frameshift) mutations, their function may also be disrupted by missense mutations in critical regions. These missense mutations, unlike hotspot mutations in oncogenes, often are not recurrent at individual positions, but instead their recurrence may only be evident in mutational clusters. By using protein structures, we identified potentially inactivating mutational clusters in critical regions of several tumor suppressors including *PTEN*, *CDH1*, and *KEAP1*.

**Fig. 3:**
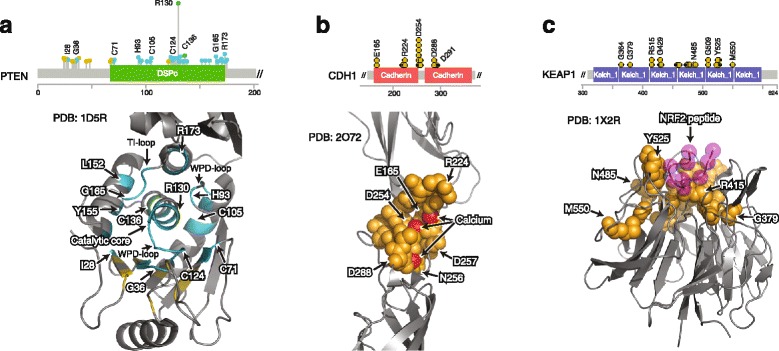
Examples of mutational 3D clusters in tumor suppressor genes. **a** Residues in 3D clusters in *PTEN* highlighted in the protein sequence (*top*) and a protein structure (*bottom*). The 3D cluster residues surround the catalytic site. **b** Residues in 3D clusters in *CDH1* (E-cadherin) highlighted in the protein sequence (*top*) and a protein structure (*bottom*). The 3D cluster mutations are likely to disrupt the critical calcium-binding site (calcium atoms in *red*). **c** 3D clusters in *KEAP1* in the protein sequence (*top*) and a protein structure (*bottom*). Most of the 3D cluster mutations are in the NRF2-binding region (NRF2 peptide in *purple*)

*PTEN* is one of the most frequently mutated tumor suppressors with mutations occurring in various cancers. In *PTEN*, we identified 15 3D clusters that included 48 residues (2 single-residue hotspots, 46 rarely mutated residues) (Fig. 3a, Additional file 3: Table S2). All these clusters reside in the flanking regions surrounding the phosphatase catalytic core motif (Fig. 3a), a region that is necessary for *PTEN* activity [29].

*CDH1* encodes E-cadherin, a transmembrane glycoprotein mainly expressed in epithelial cells. Germline mutations in CDH1 are associated with an increased risk of gastric and breast cancer [30], and CDH1 somatic inactivation via epigenetic silencing or truncating mutations is common in both cancer types. We identified 11 3D cluster residues (all rarely mutated residues; mutation frequency 0.01–0.06% individually) in CDH1 (Fig. 3b, Additional file 3: Table S2). Out of the 19 samples with these 3D cluster mutations, 11 were gastric tumors. Although distant in amino acid position (between the 165th and 291st residues), in 3D space, all of these residues surround the junction between the first and second extracellular cadherin domains in the 3D structure (Fig. 3b). Mutations in these residues are likely to perturb functionally essential calcium-binding sites in the junction region [31] and hence are likely inactivating and potentially oncogenic.

KEAP1 is a substrate adapter protein for the E3 ubiquitin ligase that targets NFE2L2 (NRF2) for ubiquitination and subsequent degradation. Loss-of-function mutations in key KEAP1 residues result in accumulation of NRF2 in the nucleus and contribute to chemoresistance in vitro [32]. We identified 26 3D cluster residues (all rarely mutated residues; mutation frequency 0.01–0.03% individually) in KEAP1 (Fig. 3c, Additional file 3: Table S2). These mutations were localized to the interaction domain of KEAP1, suggesting that they likely disrupt NRF2 binding (Fig. 3c). Notably, out of the 36 samples with these mutations, 18 were lung adenocarcinomas, 6 of which lacked hotspot mutations.

### Functional validation of rare mutations identified in 3D clusters

Identifying mutations in genes for which targeted therapies exist or are being developed, regardless of their individual frequency in the population, is critical for the effective practice of precision oncology. Our analysis identified 3D clusters in several genes for which selective inhibitors are either used as part of standard clinical management or are being actively tested in clinical trials, including *EGFR*, *KIT*, *MTOR*, *PIK3CA*, *MAPK1*, and *FGFR3* (Table 1). The 3D clusters within these genes contained known activating single-residue hotspot mutations as well as rare candidate driver mutations. While the function of most of these rare mutations is unknown, a subset has been functionally characterized in prior studies. For example, EGFR T263P has been reported to induce oncogenic EGFR activation [33], and recently, many of the rare mutations in MTOR present within 3D clusters (A1459P, L1460P, Y1463S, T1977R, and V2006I/L) (Table 1) have been shown to induce increased mTORC1/2 pathway activity [34].

**Table 1:** Example 3D clusters with potential functional targets

Gene	PDB ID: chain	Position (number of mutated samples)	*p*	Cancer types (number of mutated samples)^*^
*EGFR*	1IVO:B	R252(8) F254(1) D256(2) K261(1) T263(2) C264(1) A289(28)	0.016	GBM(30) LGG(8) Stomach ADCA(2) Other(3)
*EGFR*	2JIU:B	V769(1) R831(2) R832(2) L833(2) L858(30) L861(7) H893(1)	0.025	Lung ADCA(39) Lung SCC(2) CRC(2) Other(2)
*KIT*	4HVS:A	W557(1) V559(3) V560(1) L576(2)	0.085	Melanoma(6) Stomach ADCA(1)
*MTOR*	4JT5:B	A1459(1) L1460(2) V1461(1) Y1463(1) K1465(1) M1467(1) R1480(2) C1483(5)	0.035	ccRCC(7) BRCA(1) CRC(1) Other(5)
*MTOR*	4JSN:A	A1971(3) I1973(2) Y1974(1) T1977(3) M1998(1) V2006(2)	0.047	ccRCC(4) CLL(2) Endometrial CA(2) Other(4)
*PIK3CA*	2v1y_A	R38(14) E39(5) R88(40) C90(4) R93(15)	0.014	Endometrial CA(27) CRC(19) Other(32)
*MAPK1*	4FV5:A	E81(2) R135(1) G136(1) D321(3) E322(15)	0.014	Cervical SCC(9) HNC(9) BRCA(1) Other(3)
*FGFR3*	1RY7:B	R248(9) S249(18) P250(1) D280(2)	0.050	Bladder CA(17) HNC(6) Lung SCC(3) Other(4)

*Full cancer type names are listed in the Abbreviations section

To confirm that the method could identify functional driver mutations that would not have been nominated by previously reported frequency-based methods, we functionally tested several rare mutations identified in 3D clusters in the *MAP2K1* and *RAC1* genes. Components of the MAPK pathway are among the most commonly altered genes in human cancer. Our method revealed 3D clusters in all three RAS proteins (K/N/H-RAS), RAC1, BRAF, MAP2K1, and MAPK1 in a variety of cancer types. MEK1, which is encoded by the *MAP2K1* gene, is a dual specificity kinase that phosphorylates ERK to propagate MAPK signaling transduction. Activating mutations in MAP2K1 have been shown to result in constitutive MAPK pathway activity and to confer resistance to RAF inhibition and MEK inhibitor sensitivity [35, 36].

**Fig. 4:**
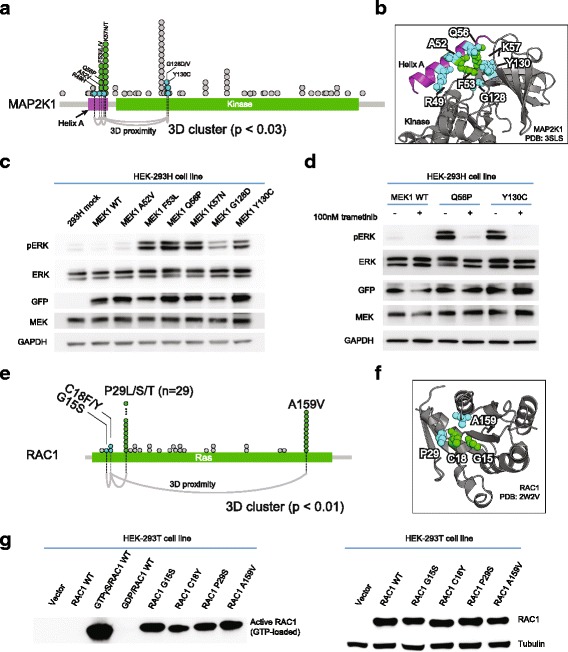
Experimental validation of functional impact of mutations in 3D clusters in MAP2K1 and RAC1. **a** Seven residues in a 3D cluster in MAP2K1, in the context of the domain structure of the protein. Notation as in Fig. 1: each *circle* is an occurrence in a sample; *connecting lines* (*bottom*) indicate cluster membership, i.e., statistically significant proximity in 3D in the protein structure. **b** The same cluster of mutated residues in the 3D structure of MAP2K1. The *purple helix* is known to negatively regulate the kinase activity of MAP2K1/MEK1. **c** Functional characterization of MAP2K1/MEK1 mutants in HEK-293H cells. Expression of G128D and Y130C (as well as the previously characterized F53L, Q56P, and K57N) mutants each resulted in increased expression of phosphorylated ERK compared to wild-type MAP2K1 — but not the cluster member A52V. **d** ERK phosphorylation was inhibited by trametinib in cells expressing the Q56P or Y130C MAP2K1 mutations in HEK-293H cells. **e** The four residues (two single-residue hotspots: P29 and A159, and two rarely mutated residues: G15 and C18) in the identified 3D cluster in RAC1 in the linear domain structure of the protein. **f** The same cluster in the 3D structure of RAC1. **g** Western blot analysis of RAC1 activation (GTP-bound RAC1 levels) by PAK1 pulldown (*left*) and of total RAC1 levels (*right*) in HEK-293 T cells. The RAC1 3D cluster mutations G15S and C18Y, as well as the previously characterized P29S and A159V, were associated with significant RAC1 activation, as compared to wild-type RAC1

We identified a 3D cluster (*p* = 0.03) in MAP2K1 that included seven mutated residues (R49, A52, F53, Q56, K57, G128, and Y130). Two of these residues (F53 and K57) are single-residue hotspots [6] and are shown to induce constitutive ERK pathway activation [37]. The other five were rarely mutated (mutation frequency of 0.01–0.03% individually) (Fig. 4a). All seven of these mutated residues reside in the shared interface between helix A and the kinase domain (Fig. 4b). As helix A has previously been shown to negatively regulate MEK1 kinase activity by interacting with the kinase domain [38], mutations that disrupt this interaction may result in constitutive ERK pathway activation. We thus experimentally assessed the ability of the mutations in this 3D cluster to induce ERK1/2 phosphorylation in a cellular model. We found that expression of five of the mutated proteins, including G128D, Y130C, and also the previously characterized F53L, Q56P, and K57N mutations [37], induced downstream MAPK signaling as assessed by increased expression of phosphorylated ERK (Fig. 4c). To test whether the Y130C variant protein that is not in a single-residue hotspot, but was nominated by 3D cluster analysis, is sensitive to MEK inhibition, we treated HEK-293 T cells expressing the Y130C mutant, or as a positive control the Q56P mutant, with trametinib, an FDA-approved MEK inhibitor. Trametinib treatment resulted in significant down-regulation of MAPK pathway activity (Fig. 4d). As durable responses to MEK inhibitors have been reported in patients whose tumors have an activating mutation in MAP2K1 [36], this example highlights the potential translational impact of 3D cluster analysis.

RAC1 is a Rho family small GTPase that has been recently implicated to confer resistance to RAF inhibition in vitro and may underlie early resistance in patients [39]. Recently, two oncogenic single-residue hotspots in RAC1 were identified, P29 and A159, both of which activate RAC1 in vitro [6]. We identified a statistically significant 3D cluster of four residues (*p* = 0.009) in RAC1, which, in addition to P29 and A159, includes novel rare mutations at amino acids G15 and C18 (mutation frequency of 0.01–0.02%, respectively) (Fig. 4e and f). To confirm that these mutations activate RAC1, we utilized a PAK1-pulldown assay to quantify activated RAC1 expression in cells expressing mutant and wild-type RAC1 protein. We found that, compared to wild-type RAC1, both the G15S and C18Y RAC1 mutants resulted in elevated active RAC1 expression (Fig. 4 g). These results expand the number of experimentally validated activating alleles in RAC1, suggesting that RAC1 G15S and C18Y mutations in this 3D cluster may possess similar biological consequences to those of the previously characterized RAC1 hotspot mutations.

In-depth functional testing of mutations in the more than 3000 potential driver alleles in 503 genes identified by our 3D cluster method could not be feasibly performed by a single laboratory. Therefore, to facilitate this effort, we have made publically available all the mutations revealed by this analysis via an interactive website, http://3dhotspots.org. On the website, users can view and search for mutations in 3D clusters, explore details about each mutation and cluster, and visualize the mutations in interactive 3D structures (Fig. 1b). Mutations that are part of a 3D cluster will also be highlighted in all mutation tables in the cBioPortal for Cancer Genomics, with a link to http://3dhotspots.org (Fig. 1b). We intend to keep the site up to date as additional mutational and protein structure data are generated. We anticipate that these data will provide a basis for detailed biological studies by investigators with gene-specific expertise and could also be used to guide clinical trial eligibility for molecularly driven studies in precision medicine.

### Comparison of 3D hotspot detection tools

Alternative, independently developed 3D cluster detection methods have also identified recurrent mutations that cluster in 3D structures. All of these methods evaluate recurrence as occurrence above a statistical random background, counting occurrences of a mutation in any member of a 3D cluster in a set of tumor samples. However, the methods differ in detail, e.g., in the tumor sets analyzed, the definition of 3D clusters, and the statistical test applied, and so they produce different lists of candidate functional mutations. For example, Mutation3D identified 399 mutated residues in 75 genes as likely functional [17], HotMAPS identified 398 mutated residues in 91 genes [18], and Hotspot3D identified 14,929 mutated residues in 2466 genes [19], whereas our method identified 3404 mutated residues in 503 genes (Additional file 6: Table S5 and Additional file 7: Figure S2). Somewhat surprisingly, only 15 mutated residues were identified by all four methods, all of which were also previously identified as single-residue hotspots [6]. Of the 3404 mutated residues, 2908 detected by our method were not identified by any of the other three methods, including MAP2K1 Q56 and K57, which we experimentally validated. Comparison to a recent experimental in vivo screening study of rare mutations by Kim et al. [20] also confirmed that the four methods have different coverage and power to detect rare driver mutations and therefore provide complementary datasets of candidate functional mutations (Additional file 8: Table S6). For example, the method described here was able to detect the KRAS D33E and SPOP K134N mutations that were validated as functional by Kim et al. [20], but the other three methods did not detect these mutations as statistically significant.

## Discussion

Tremendous effort has been invested in the discovery of therapeutic agents to suppress oncogenic signaling. These efforts have resulted in several FDA-approved agents that target a variety of genes and pathways in several different cancer types. For instance, vemurafenib, a selective inhibitor of *V600E/K mutant BRAF*, was first approved in metastatic melanoma, a cancer in which approximately 50% of tumors harbor a *BRAF V600E/K* mutation [40]. Vemurafenib has since shown activity in a wide spectrum of malignancies that share this actionable mutation [41], suggesting that molecular biomarkers can be predictive of drug response across cancer types. However, effective development and use of targeted therapies necessitate identification of “driver” mutations among the far more prevalent passenger mutations in patient genomes. Many of these mutations can be identified by their recurrence in a single position, but others are less common or private to a particular tumor. One property they often share with single-residue hotspots and previously functionally characterized mutations is 3D proximity; i.e., rare mutations can be physically close to each other or to a known and common mutation in the same protein, raising the possibility that these mutations are also driver events. To prioritize rare driver mutations for functional or clinical validation, we developed a novel method that identifies significantly mutated regions in 3D protein structures. We applied this method to more than 11,000 tumors analyzed by whole exome or genome sequencing.

Our analysis identified several thousand, mostly novel, candidate functional cancer mutations. While some mutations in the 3D clusters were in single-residue hotspots, which by definition are frequently mutated in cancer, the majority were rare mutations. Functional annotation is often not available or sparse for these rare mutations. On the one hand, rarely mutated residues coupled to a single-residue hotspot often occur in many well-studied oncogenes (such as *KRAS*, *BRAF*, *EGFR*, *PIK3CA*, and *MTOR*, among many others) and in several frequently mutated tumor suppressor genes (such as *TP53* and *PTEN*). It is plausible that the functional impact of such mutations is similar to those in the single-residue hotspots, and hence transfer of functional annotation from the common mutations to the rare mutations in the same 3D cluster makes sense. On the other hand, the functional annotation of rarely mutated residues, which are not coupled in a 3D cluster to a single-residue hotspot but instead clustered only with other rarely mutated residues, is much less certain. Fortunately, the placement of the clusters of mutated residues in known 3D structures affords the opportunity for informative mechanistic hypotheses facilitating the design of focused functional studies. For example, we identified a cluster of mutations that likely disrupt critical calcium-binding sites in *CDH1*, a tumor suppressor that mediates cell adhesion. Another example is a cluster of mutations in *KEAP1* that potentially disrupt binding sites with NRF2, a key regulator of the cellular oxidative response.

By experimentally validating candidate functional mutations in 3D clusters in MAP2K1 and RAC1, we show that our method readily identifies previously occult rare activating mutations that could not be revealed by positional frequency analyses alone and that a subset of such mutations are potential biomarkers of sensitivity to targeted inhibitors in individual patients with cancer. We showed, for example, that the rare MAP2K1 G128D and Y130C mutations induce MAPK pathway activation and that such mutations retain sensitivity to MEK inhibitor treatment in vitro. While some mutations identified by our analysis were not activating in vitro, such as MAP2K1 mutations of A52, by analyzing mutations in the context of protein structures, we can form hypotheses about the biochemical reasons for such results: in this case, A52 does not interact strongly with the kinase domain in the wild-type 3D structure (Fig. 4b). This example illustrates the potential functional insights resulting from detailed analysis of individual cancer mutations in the context of 3D structures.

A proportion of rare mutations are not only biologically interesting (since they potentially promote tumor initiation or progression), but also clinically important with the advent of genomic-based clinical trial designs (such as the NCI-Molecular Analysis for Therapy Choice (NCI-MATCH) trial). Forty-five percent of the 11 K tumor samples in our dataset lacked a single-residue hotspot driver mutation, and identifying the genetic drivers of these patients is a critical step for the choice of therapy, design of clinical trials, or drug development. Here, we achieved a partial advance in this direction by identifying potential driver mutations in 17% of the samples without single-residue hotspot driver mutations (8% of all samples). Some of the identified mutations, e.g., those in *MTOR*, *EGFR*, and *MAP2K1*, could have immediate translational importance. For example, clinical trials enrolling patients with MAPK pathway mutations, e.g., the NCT01781429 trial, could expand their eligibility criteria beyond single-residue hotspot mutations in the MAPK pathway and enroll patients with the MAP2K1 3D cluster mutations identified here.

While our approach can identify novel and potentially interesting mutations in cancer genes and in genes previously unknown to be involved in cancer, the method is still limited by the lack of complete protein structure data for many genes. For the 18,100 genes with mutations in our dataset, we were able to align 7390 of them to one or more protein structures. However, for many genes, the structures included only individual protein domains, limiting the scope of our analysis. There were only 1307 genes with a protein structure that covered more than 90% of the protein length, and only 3183 genes with more than 50% coverage. This limits the ability of our algorithm to detect 3D clusters that were not close in sequence, for example, those involved in domain-domain interactions. Fortunately, as protein structure characterization technologies such as cryo-electron microscopy (cryo-EM) advance, more protein structures, and more complete protein structures, are being generated. We can also make use of the remarkable progress in 3D protein structure prediction using evolutionary couplings for proteins that are members of protein families with many known homologous sequences (http://evfold.org) [42, 43]. We thus plan to periodically include new protein structures in our analysis pipeline, which along with the inclusion of additional sequencing data will allow for the nomination of additional novel 3D clusters. Given the current coverage of human proteins by 3D structural knowledge, one can expect a steady increase in the number of candidate functional mutations identified by methods of this type as more accurate structures of most human proteins become available.

Like any statistical method, the power of our approach is also limited by the number of available tumor samples. For example, a 3D cluster in AKT1 (R15, E17, W22, and D323) did not score as statistically significant (*p* = 0.11) as a 3D cluster. There is no issue with the fact that the cluster contains the most frequent single-residue hotspot mutation E17K, which has been evaluated as an indicator of response to AKT-targeted inhibitors in clinical trials [44]. But D323 is not identified as a candidate by our method on the current dataset, while experimental in vitro studies indicate that AKT1 D323 mutations lead to constitutive activation of AKT [45]. Fortunately, as more cancer genomic data are generated, additional significant 3D clusters will likely emerge.

We have shown that the mutational 3D clusters identified by three alternative methods (Mutation3D [17], HotMAPS [18], and Hotspot3D [19]) and our method are largely complementary (Additional file 7: Figure S2). While different mutational and structural datasets used by these four tools may have led to some of the differences observed, methodological differences likely dominate. For example, unlike the other methods, HotMAPS identified some single-residue hotspots as functional without clustering them with other residues in 3D structures; e.g., IDH1 R132 was predicted by HotMAPS as the only recurrently mutated residue in the gene. Another methodological difference was the distance cutoffs that were used to decide whether two residues are interacting in 3D structures. For example, Hotspot3D utilized interactions of longer distance (comparing to other methods), e.g., IDH2 R172 was detected in a cluster with R140 with a distance of 10 Å. Another reason for differences in results from the different methods may be due to differences in the sensitivity and specificity levels. Mutation3D and HotMAPS used a high-specificity and low-sensitivity cutoff and therefore predicted as functional only about 400 mutated residues in less than 100 genes, most of which were single-residue hotspots. Conversely, Hotspot3D nominated close to 15,000 mutated residues in almost 2500 genes (potentially high sensitivity), which may include many false positives (low specificity). An analysis of the results of a pooled in vivo tumor formation assay and gene expression profile of numerous low frequency somatic genetic variants by Kim et al. [20] supports this observation: All mutations identified by Mutation3D and most mutations identified by HotMAPS that were shown to be functional in the screen were single-residue hotspots, whereas our method and Hotspot3D were able to identify significantly more of the functional rare mutations. Finally, the Hotspot3D prediction included a considerable number of false positives (false detection rate 32% compared to 12% of our method when applied to the Kim et al. data) (Additional file 8: Table S6). As there is no definitive comprehensive gold standard of mutations with positive functional impact for the proliferation of cancer cells, it is reasonable to take the top-ranked results of any of the available methods as a point of departure for functional genomics experiments, while taking into consideration the qualitative differences between the different methods.

## Conclusions

In this work, we present a novel computational method for identifying mutational 3D clusters of potential functional significance with results based on the largest whole exome or genome dataset analyzed in the context of protein structures to date. We identified putative driver mutations in more than 3000 protein residues, the majority of which are rare mutations that have not been identified by previous gene-, residue-, or cluster-based methods of recurrence analysis. We experimentally validated an activating role of a few rare mutations in MAP2K1 and RAC1 as a proof of concept that computational 3D structure analysis of mutations can generate useful hypotheses for functional and preclinical validation.

By making regularly updated results available through an interactive website (http://3dhotspots.org) as well as via the widely used cBioPortal for Cancer Genomics, we hope to facilitate future functional and clinical testing of numerous candidate driver alterations, with increasing accuracy as larger datasets become available. While large-scale unbiased experimental screening has proven to be successful in identifying novel functional mutations in cancer [20], our results provide a way to prioritize variants and have the potential to considerably increase the efficiency of functional screening experiments. This work has immediate translational significance, as it can potentially be used directly to help guide clinical trial enrollment of patients based on individual tumor profiles.

## Additional files

Additional file 1: Figure S1. Illustration of the permutation procedure for calculating the statistical significance of 3D clusters. (PDF 327 kb)
Additional file 2: Table S1. All identified 3D clusters. (XLSX 114 kb)
Additional file 3: Table S2. All residues of their classes of the identified 3D clusters. (XLSX 122 kb)
Additional file 4: Table S3. The numbers of 3D cluster residues per gene by residue class. (XLSX 24 kb)
Additional file 5: Table S4. The fraction of samples with 3D clusters per gene by residue class. (XLSX 27 kb)
Additional file 6: Table S5. Significantly mutated residues identified in 3D structure clusters by our method and three previous methods (Mutation3D, HotMAPS, and Hotspot3D). “x” indicates a residue was identified in a mutational cluster in a 3D structure by the corresponding method. (XLSX 350 kb)
Additional file 7: Figure S2. Comparison of mutated residues identified in 3D structure clusters by our method and those by three alternative methods (Mutation3D, HotMAPS, and Hotspot3D). (PDF 324 kb)
Additional file 8: Table S6. Comparison of four *in silico* methods for detecting potential driver mutations in 3D structures to the in vivo experimental screen by Kim et al. Cancer Discovery 2016. “x” indicates a residue was identified in a mutational cluster in a 3D structure by the corresponding method. “-” indicates it was not. *Green* a functional mutation by Kim et al. also detected in a 3D mutational cluster, *yellow* a functional mutation by Kim et al. *not* detected in a 3D mutational cluster, *red* a neutral mutation by Kim et al. detected in a 3D mutational cluster. (XLSX 11 kb)

## Abbreviations

3D: Three-dimensional
ACC: Adrenocortical carcinoma
ACyC: Adenoid cystic carcinoma
ADCA: Adenocarcinoma
ALL: Acute lymphoid leukemia
AML: Acute myeloid leukemia
BL: Burkitt lymphoma
BRCA: Breast carcinoma
CA: Carcinoma
ccRCC: Clear cell renal cell carcinoma
chRCC: Chromophobe renal cell carcinoma
CLL: Chronic lymphoid leukemia
CRC: Colorectal carcinoma
cSCC: Cutaneous squamous cell carcinoma
DLBCL: Diffuse large B-cell lymphoma
GBM: Glioblastoma
HCC: Hepatocellular carcinoma
HGG: High grade glioma
HGSOC: High grade serous ovarian cancer
HNC: Head and neck carcinoma
ICGC: International Cancer Genome Consortium
JPA: Juvenile pilocytic astrocytoma
LGG: Low grade glioma
MBL: Medulloblastoma
MCL: Mantle cell lymphoma
MDS: Myelodysplasia
MM: Multiple myeloma
MMMT: Uterine carcinosarcoma
MRT: Rhabdoid cancer
NPC: Nasopharyngeal carcinoma
PCa: Prostate adenocarcinoma
PNET: Primitive neuroectodermal tumor
pRCC: Papillary renal cell carcinoma
SCC: Squamous cell carcinoma
SCLC: Small cell lung cancer
TCGA: The Cancer Genome Atlas
