# Supplementary material for: 3D clusters of somatic mutations in cancer reveal numerous rare mutations as functional targets
Source: Genome Med. 2017 Jan 23;9:4. doi: 10.1186/s13073-016-0393-x (PMC5260099; doi:10.1186/s13073-016-0393-x)
Supplement: Additional file 1: Figure S1. — Illustration of the permutation procedure for calculating the statistical significance of 3D clusters. (PDF 327 kb) [file 13073_2016_393_MOESM1_ESM.pdf]

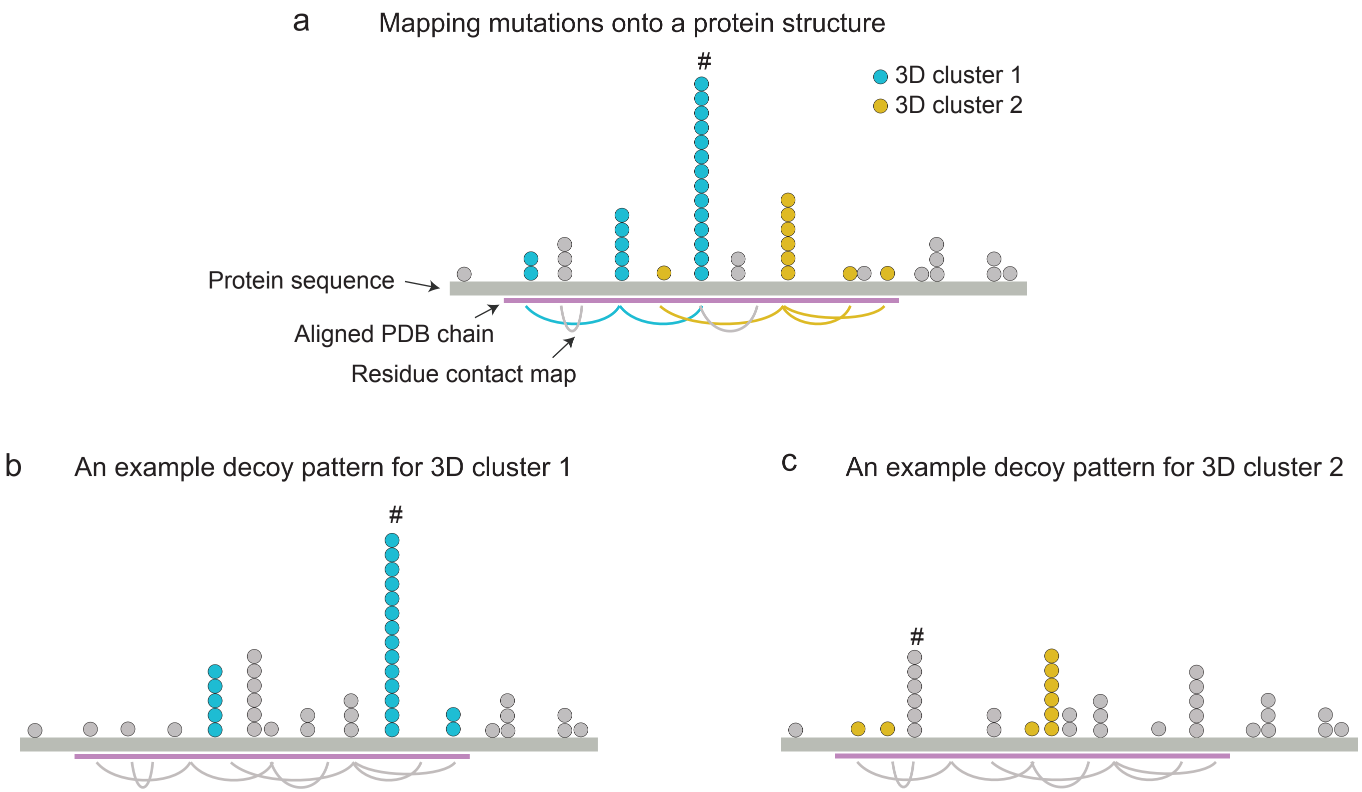
a
Mapping mutations onto a protein structure
#
3D cluster 1
3D cluster 2
Protein sequence
Aligned PDB chain
Residue contact map
b
An example decoy pattern for 3D cluster 1
#
c
An example decoy pattern for 3D cluster 2
#
