# Supplementary material for: 3D clusters of somatic mutations in cancer reveal numerous rare mutations as functional targets
Source: Genome Med. 2017 Jan 23;9:4. doi: 10.1186/s13073-016-0393-x (PMC5260099; doi:10.1186/s13073-016-0393-x)
Supplement: Additional file 7: Figure S2. — Comparison of mutated residues identified in 3D structure clusters by our method and those by three alternative methods (Mutation3D, HotMAPS, and Hotspot3D). (PDF 324 kb) [file 13073_2016_393_MOESM7_ESM.pdf]

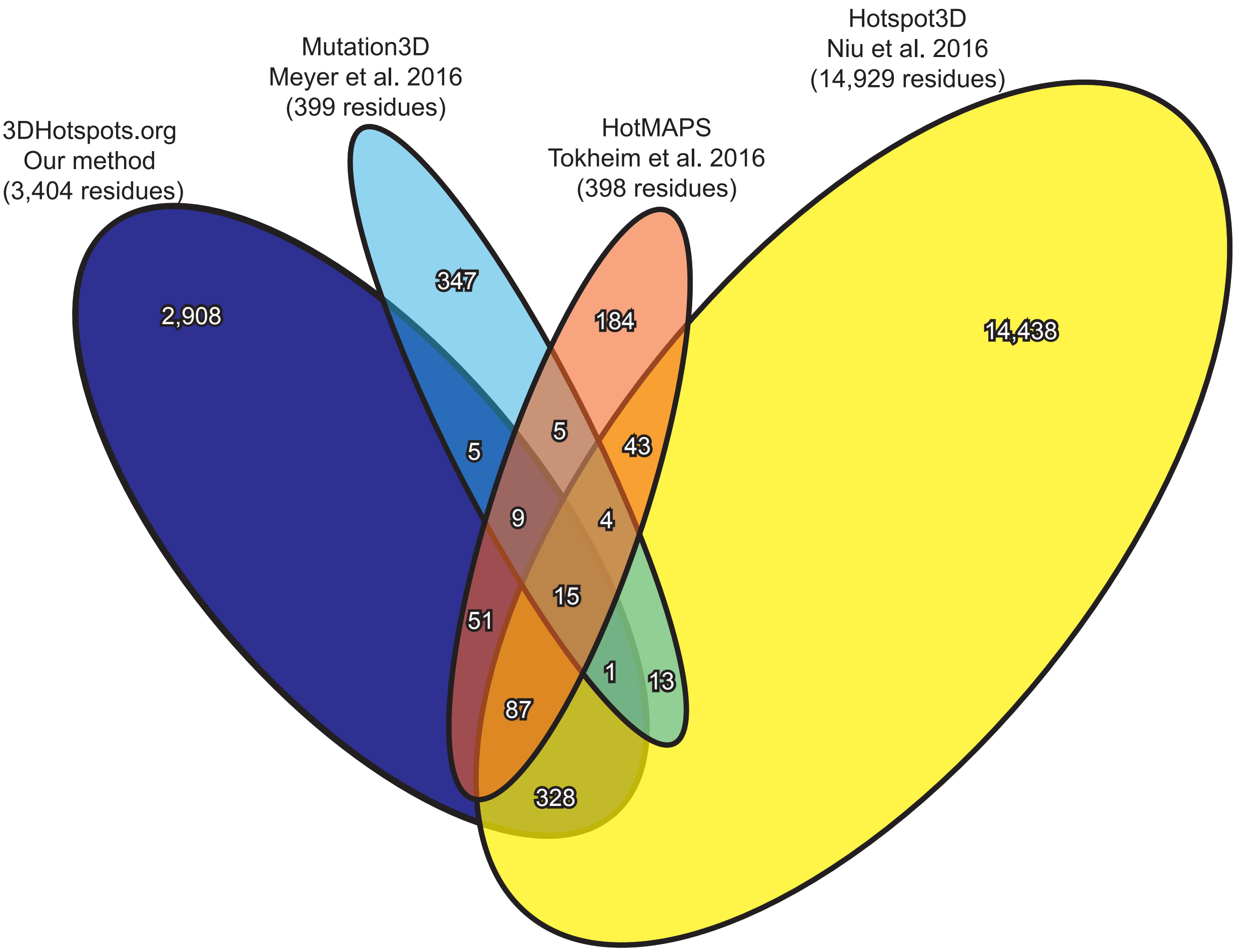
3DHotspots.org
Our method
(3,404 residues)
Mutation3D
Meyer et al. 2016
(399 residues)
HotMAPS
Tokheim et al. 2016
(398 residues)
Hotspot3D
Niu et al. 2016
(14,929 residues)
2,908
347
184
14,438
5
5
43
9
4
15
51
1
13
87
328
